# Factors Affecting Perceived Health Benefits and Use Behaviors in Urban Green Spaces During the COVID-19 Pandemic in Southern China Megacities

**DOI:** 10.3389/fpubh.2021.759444

**Published:** 2021-10-28

**Authors:** Haiwei Li, Weijing Luo, Yongqi Hou, Yu Xia, Jing Yao, Ning Kang, Congshuang Deng, Hu Sun, Chongxian Chen

**Affiliations:** ^1^College of Forestry and Landscape Architecture, South China Agricultural University, Guangzhou, China; ^2^Urban Big Data Centre, School of Social and Political Sciences, University of Glasgow, Glasgow, United Kingdom; ^3^School of Architecture, Tsinghua University, Beijing, China; ^4^Guangzhou Sun & Partners Incorporation Design Co., Ltd., Guangzhou, China

**Keywords:** green spaces, health, use behaviors, place attachment, citizens

## Abstract

**Background:** The COVID-19 pandemic has alienated people from urban green spaces (UGSs) that have various health outcomes for humans. However, little is known about the influential factors of perceived health benefits and use behaviors in UGSs during the COVID-19 pandemic. This study aims to explore the key factors that influence perceived health benefits and use behaviors in UGSs and to assess the mediating role of place attachment in relationships during the COVID-19 pandemic in Chinese megacities.

**Methods:** We conducted an online questionnaire survey from December 2020 to March 2021 in Guangzhou and Shenzhen, China. Six multiple regression models were constructed to investigate the main factors by which UGSs influence citizens' perceived health benefits and use behaviors. Four mediation models were established using the structural equation modeling (SEM) method to explore the mediating effect of place attachment.

**Results:** A total of 628 questionnaires were included in the analysis. The results revealed that some UGS components (green space access, maintenance, and soundscape) significantly affected perceived health benefits for citizens (physical, mental, and social health) during the COVID-19 pandemic. Conversely, use behaviors (frequency of visits, duration of visits, and activity intensity) were mainly affected by the sociodemographic context but less affected by UGS components. In addition, UGS components were found to significantly predict place attachment, which in turn influenced the perceived health benefits, frequency, and duration of visits.

**Conclusions:** This study distinguished the key factors that affect perceived health benefits and use behaviors during the COVID-19 pandemic: green space access, maintenance, soundscape, and sociodemographic characteristics. Place attachment still needs to be considered when discussing how to encourage citizens to visit UGSs during the pandemic. These findings provide implications for policymakers and landscape planners regarding design and management measures for UGSs that are conducive to coping with pandemics.

## Introduction

COVID-19 has caused unprecedented disruption to human health and well-being worldwide (1). Isolation measures have further alienated people from urban green spaces (UGSs), changing their perceptions and use behaviors of these spaces (2) after the World Health Organization (WHO) declared COVID-19 a public health emergency on 30 January 2020 (3). Moreover, the drastic changes produced by rapid urban sprawl in megacities make residents feel a lack of connection with UGSs. Unhealthy lifestyles, including a reduction in outdoor activities involving contact with nature, have further deteriorated people's health (4). Green infrastructure, a network composed of different types of green spaces (5), has been proven to have many benefits (6) and has been one of the most vital and effective infrastructures to improve health in response to previous pandemics (7). Therefore, exploring how people perceive the relationships between UGSs and health of great interest for researchers and policymakers to improve people's visitation of UGSs for health purposes in the context of the normalization of the pandemic and urbanization.

Previous studies have demonstrated that exposure to UGSs is beneficial for residents' health. UGSs may exert an influence on positive health outcomes, such as lower rates of heart disease, stroke, obesity, stress, and depression (4), by encouraging physical activity (8), relieving mental fatigue (9, 10), and facilitating social interaction (11). Numerous studies during non-pandemic periods have confirmed that the sociodemographic context, individual perceptions of UGSs, and the perceived benefits of UGSs may affect UGS visitation and human health (8, 12). Green spaces with diverse characteristics influence people's use experiences and perceptions and thus influence their use behaviors, such as visiting duration and frequency (12, 13). In turn, improving people's green space utilization increases perceived health benefits (11). Psychological factors are also worth noting (14–16). For example, studies have shown the potential of place attachment to contribute to perceived health benefits and behavioral intentions, including promoting positive emotion, enhancing social cohesion, and raising people's awareness of environmental protection (17, 18). Recently, an increasing number of studies have turned their attention to the relationships among changes in perceptions of UGSs, use behaviors, and human health during the COVID-19 pandemic (19–21). Nevertheless, there is no consensus on the most influential factors for perceived health benefits and use behaviors in UGSs during the COVID-19 pandemic, especially in megacities in China.

In this study, we investigate the UGS components that influence perceived health benefits and people's use behaviors to further explore the role of place attachment in these relationships in Guangzhou and Shenzhen, China, during the COVID-19 outbreak. The objective of our research is to study the mechanisms underlying these associations and provide suggestions for policymakers and landscape planners to design and manage UGSs in ways that are more conducive to coping with the pandemic.

## Literature Review and Theoretical Framework

### Perceived Health Benefits and Green Spaces

Based on the health belief model theory, perceived health benefits refer to individuals' perceptions of the positive changes that result from a particular action to reduce the threat of illness (22). In turn, these positive changes influence people's attitude toward their external environment (23). Thus, the perceived health benefits of UGSs may reflect people's attitude toward visiting UGSs and influence their actual health.

Numerous studies have demonstrated that UGSs were perceived as a preventative infrastructure for human health before pandemics or crises (7). For example, a study in the UK found that residents with existing health problems particularly acknowledged the benefits of gardening, and the health benefits increased their frequency of gardening (24). A study in China also demonstrated that visitors' perceived restoration and mental health from some green spaces directly affect their visit intentions (25). However, during the COVID-19 pandemic, the continuous spread of the coronavirus disrupted people's health, bringing about fear and anxiety (26), reducing physical activity (27), and causing a loss of connection with others in outdoor environments (28). Moreover, public space shutdowns have changed people's perceptions of UGSs. For instance, people who perceived greater accessibility of private gardens had a greater protective effect during the first COVID-19 peak, but this effect diminished in the post-peak period (29). In addition, many studies have demonstrated the role of perceived health benefits in behavioral intention regarding green spaces (30–32). For example, some researchers have noted that perceived health benefits have a positive impact on people's willingness to protect (33) and usage of green spaces (31). However, relevant discussions in the context of the COVID-19 pandemic are still limited. Therefore, it is increasingly necessary to investigate how people perceive UGSs and their health benefits during the pandemic.

### Use Behaviors and Green Spaces

People's use behaviors of UGSs often refer to usage frequency, duration, and activity intensity (12, 13, 34). A growing body of research in non-pandemic periods suggests that the physical features of green space influence people's use behaviors. For instance, green space size (35), proximity (36), maintenance (12), facilities (37), and aesthetic features such as vegetation (38), water (12), and sound (39) are physical factors that are closely related to green space visitation. Giles-Corti et al. suggested that larger green spaces are related to more physical activity (40). Trees, water, and natural sounds that are recognized as salutogenic features have also been found to play a key role in determining people's use behaviors (41). In addition, some researchers have suggested that the way people perceive the environment and their satisfaction with the quality of green spaces, including perceived greenery and accessibility, are interrelated with park use patterns (42, 43). However, people's use behaviors of UGSs have changed during the COVID-19 pandemic. For example, more physical activity has taken place in small nearby urban green spaces rather than in larger but further spaces during the COVID-19 pandemic, and visiting green spaces has changed from unnecessary for residents in the non-pandemic period to essential during the pandemic (19). Venter et al. found that increases in recreational activity were sustained for 6 months mainly in protected and cultural landscape areas in Oslo, Norway (44). However, two recent studies showed that UGS usage decreased in some European and North American cities (45), while it increased in some Asian cities (20). Clearly, studies on people's use behaviors of UGSs during the COVID-19 pandemic are limited, and the key factors affecting people's use of UGSs in different countries during the pandemic remain unclear.

### Place Attachment

Since the 1970s, many scholars have been concerned with the features of places in generating a sense of belonging and constancy (46–48). In 1974, Kasarda and Janowitz proposed the term “place attachment,” although there is no agreed-upon definition of this term. Currently, the most common concept of place attachment in environmental psychology involves the strong emotional bonds between individuals and locations that are emotionally important to them (49). Many studies have described place attachment as involving two constructs: place identity and place dependence (50, 51). Place identity reflects a personal relationship with the objective place or environment; it refers to a sense of self-extension to the site (52) and contributes to individual and social identities (53). It defines complex emotional bonding with respect to personal attitudes, thoughts, values, beliefs, meanings, and behavioral tendencies regarding the environment (54). In contrast, place dependence concerns the features and situations of a setting that satisfy an individual's needs and goals (55). Place dependence reflects how well the setting meets personal needs by cultivating emotional bonds and supporting activities through comparisons with other settings (56). These two dimensions should be considered in parallel in the assessment of place attachment.

Numerous studies have confirmed that place attachment is linked to people's perception of health benefits and behaviors (50, 54) in non-pandemic periods. Previous studies have reported that individuals' positive feelings may originate from the psychological interaction of their expectations and perceptions (57). People's expectations and perceptions of blue and green spaces determine their health and well-being to some extent (58). Place attachment represents one's sense of place and relates to a person's expectations and perceptions of a place. Thus, developing place attachment, such as forming social connections and creating meaning related to green spaces, may boost perceived health benefits. For example, an increasing number of studies have suggested that place attachment positively predicts psychological well-being, including restorative perceptions (57) and cognitive capacity (59). Depending on people's intrinsic motivation, visits to a place reflect the relationships between them and the site. For instance, two studies verified that place attachment supports people's use behaviors of small nearby parks by providing positive feelings (60) and health benefits (44). In developing a sense of place identity, place dependence, and place attachment, relationships between people and places become more extensive (61). With distinctive features that meet people's functional needs, a site can be irreplaceable and revisited (62). Clearly, place attachment plays an important role in the relationship between the environment, human health, and behaviors.

**Figure 1:**
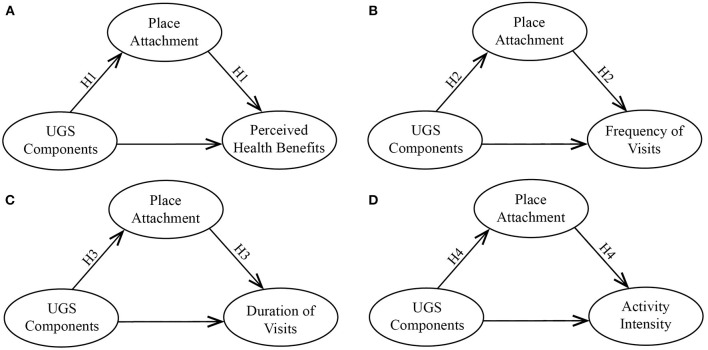
Potential relationships between UGSs, place attachment, and use behaviors. **(A)** Possible causal relationships between UGSs, place attachment, and perceived health benefits. **(B)** Possible causal relationships between UGSs, place attachment, and frequency of visits. **(C)** Possible causal relationships between UGSs, place attachment, and duration of visits. **(D)** Possible causal relationships between UGSs, place attachment, and activity intensity.

However, the perceived risk of health-related crises can destroy people's favorable perceptions of and visiting intentions for a place (63). Previous studies have shown that some pandemics in the past discouraged people from visiting places for perceived health benefits by undermining their confidence in the safety of the destinations (63, 64). Thus, the role of place attachment in the relationships among UGSs, perceived health benefits, and use behaviors is likely to be highly impacted by the COVID-19 pandemic. Moreover, little research has focused on these relationships during the COVID-19 pandemic. Thus, to confirm these inferences, we propose the following hypotheses (Figure 1):

**Hypothesis 1 (H1):** Place attachment potentially mediates the relationship between UGS components and perceived health benefits during the COVID-19 pandemic.
**Hypothesis 2 (H2):** Place attachment potentially mediates the relationship between UGS components and the frequency of visits during the COVID-19 pandemic.
**Hypothesis 3 (H3):** Place attachment potentially mediates the relationship between UGS components and the duration of visits during the COVID-19 pandemic.
**Hypothesis 4 (H4):** Place attachment potentially mediates the relationship between UGS components and activity intensity during the COVID-19 pandemic.

**Figure 2:**
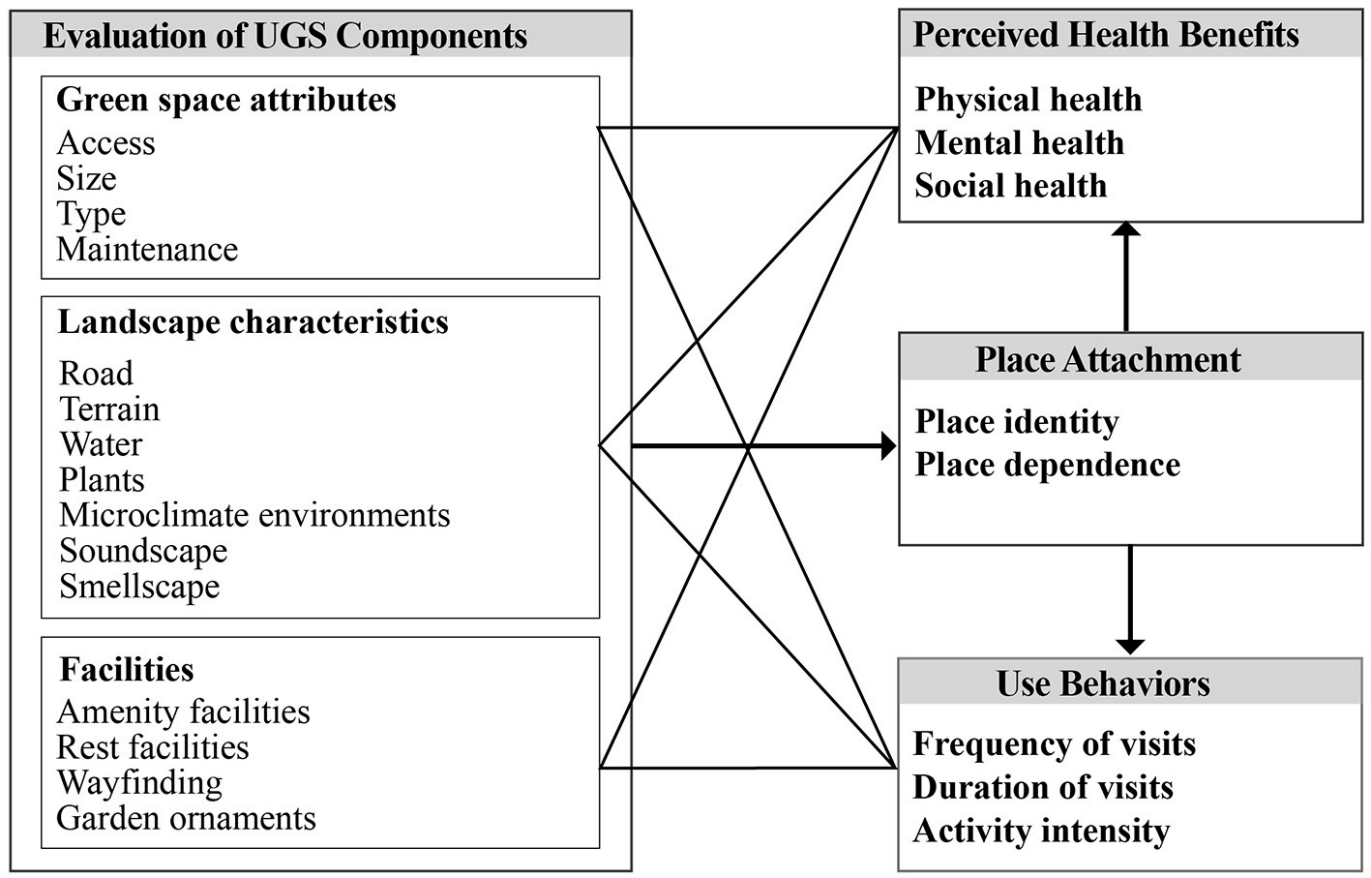
Theoretical framework of the study.

According to the literature review above, we developed the theoretical framework shown in Figure 2 for the whole study. This framework demonstrates how the UGS components affect perceived health benefits and use behaviors as well as the mediating role of place attachment in these associations to address the following questions:

1) What are the associations between UGS components and people's perceived health benefits during the COVID-19 pandemic?
2) What are the associations between UGS components and people's use behaviors during the COVID-19 pandemic?
3) Does place attachment mediate the relationships between UGS components and perceived health benefits and between UGS components and use behaviors during the COVID-19 pandemic?

Answering the above questions is beneficial to clarify the associations of UGS components, place attachment, perceived health benefits, and use behaviors among citizens, thus providing helpful information on design and management measures for policymakers and landscape planners to encourage UGS visits and promote health during the COVID-19 pandemic.

## Materials and Methods

### Study Areas and Participants

**Figure 3:**
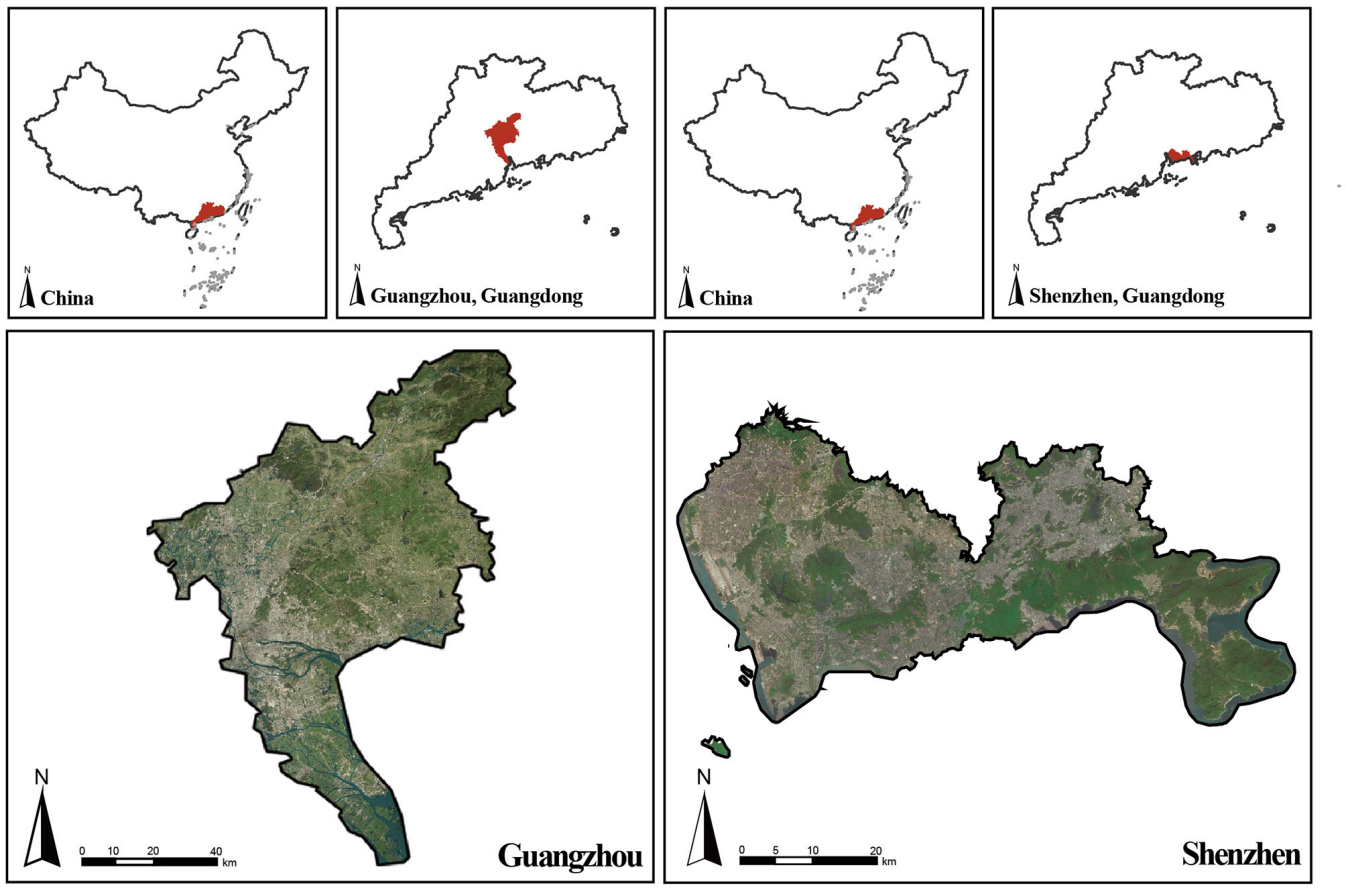
Location of the study area.

We conducted the study in Guangzhou and Shenzhen (Figure 3), two pioneering, high-density cities located in Guangdong Province in the Pearl River Delta and the Greater Bay Area. They are two of the most representative megacities and are important transportation hubs in southern China that face high risks from the COVID-19 pandemic. Guangzhou, the capital city of Guangdong, is regarded as the political, economic, and technological center of Guangdong Province and southern China. Estimates from 2019 suggest that over 15.30 million people inhabit the city's 7,434.4 km^2^ area, including 11 districts. Shenzhen, the first special economic zone in China, is an international information communications technology hub with many high-tech industries. By 2019, with 13.44 million permanent people, Shenzhen occupied a total area of 1,997.47 km^2^ and included 11 districts. With an urbanization rate of 86.46%, the vegetation in the downtown, suburban and outer suburbs of Guangzhou has been strongly disturbed, and the vegetation coverage rate has been decreasing continuously (65). By the end of 2019, Guangzhou had 247 public parks covering 5,198 hectares with a per capita public green area of 17.96 m^2^. Although Shenzhen has 1,090 public parks covering 20,077 hectares, between 2014 and 2019, the per capita public green areas decreased from 16.80 to 14.90 m^2^. Drastic urban expansion and land use changes have caused heavy chronic disease burdens for the two cities in recent years (66). Additionally, from January 2020 to March 2020, the local government imposed severe restrictions on epidemic prevention and control (20), which may have changed local people's views and habits of using UGSs. Although the emergency response level of COVID-19 prevention and control has been adjusted from level two to level three since May 2020, prevention and control of the pandemic have become normal in both cities (67). These cities' economic status, demographics, green space distribution, and special significance for the COVID-19 pandemic make them ideal places to study the associations among UGSs, perceived health benefits, use behaviors, and place attachment.

We conducted a structured online questionnaire after the first COVID-19 outbreak was effectively controlled in these two cities from December 2020 to March 2021 on the Wenjuanxing website (https://www.wjx.cn/), a convenient and frequently used online data-collection platform in China. During the COVID-19 pandemic, it has been difficult to conduct a field investigation in the affected areas in China. An online survey provided a cost-effective way to quickly investigate in different regions and reduce the risk of exposure to COVID-19. Through social media, questionnaires were randomly sent to potential participants. For this study, we limited the participants to those aged 18–65 years old and those who were likely to have access to the internet and benefit from green spaces. The participants were also told that they must answer the questions based on their perceptions during the COVID-19 pandemic and must have lived in Guangzhou or Shenzhen for more than 6 months. All information was guaranteed to be kept anonymous and used for academic research purposes only.

### Survey Instrument

The questionnaire included five sections: (1) demographic information (five questions on gender, age, education level, income, and place of residence); (2) perception of the quality or quantity of UGS components (15 questions); (3) perceived health benefits (3 questions); (4) use behaviors (3 questions); and (5) place attachment (10 questions). We developed the questionnaire based on valid, reliable studies. It contained a total of 36 questions and took ~10 min to complete.

#### UGS Components

The UGS components included green space attributes (four items), landscape characteristics (seven items), and facilities (four items). The three constructs were key factors that were likely to affect people's use of green spaces. We measured green space attributes, which describe how people perceive the accessibility, scale, types, and management of the green space with four statements: “My frequently used urban green spaces are not far from me and are easily accessible,” “My frequently used urban green spaces are very large (e.g., very small = <1 hectare and very large = more than 10 hectares),” “My frequently used urban green spaces have many different multifunctional spaces types (e.g., rest area and activity square),” and “My frequently used urban green spaces are well-maintained and managed (e.g., clean and unbroken facilities).” Landscape characteristics were measured by asking people about their perception of the quality or quantity of roads, terrain, water, plants, microclimate environments, soundscapes, and smellscapes. Facilities were also evaluated by asking how people perceived the quality or quantity of amenity facilities (e.g., seesaws, swings, and slides), rest facilities (e.g., seats and lawns), wayfinding (e.g., guide board and map), and garden ornaments (e.g., sculpture and rockery). Participants rated their level of agreement with the description of UGS components on a five-point Likert scale (from 1 = strongly disagree to 5 = strongly agree).

#### Perceived Health Benefits

Perceived health benefits are one of the main outcome variables in this study. Health is the absence of disease or infirmity and a state of complete physical, mental, and social well-being (68). Therefore, we applied three items to measure people's perceptions of health benefits, including physical (e.g., somatic function, blood pressure, and heart rate), mental (e.g., emotional regulation, attention recovery, and stress coping), and social (e.g., interpersonal relationship management, interaction, and collaboration) health benefits. We assessed the three items with the question “Do you think urban green spaces can give you physical/mental/social health benefits?” Participants rated their level of agreement on a five-point scale (from 1 = strongly disagree to 5 = strongly agree).

#### Use Behaviors

Green space use behaviors are the other main outcome variable of this study. We used three items to measure this construct: frequency of visits, duration of visits, and activity intensity. First, participants reported how often they visited UGSs on a five-point scale (1 = not at all, 2 = a few times a year, 3 = about once a month, 4 = about once a week, 5 = almost daily). Then, participants indicated their average duration of visits on another five-point scale (1 = <30 min, 2 = 0.5–1 h, 3 = 20–30 min, 4 = more than 30 min). Finally, activity intensity was measured with an item that queried participants about their activity level on a five-point scale (1 = sedentary, 2 = lightly active, 3 = moderately active, 4 = active, 5 = very active).

#### Place Attachment

We used the Place Attachment Scale (PAS) developed by Williams and Vaske (69) to determine the extent of participants' sense of the parks they frequently visited. A 10-item revised version of the Chinese PAS was used in this research. The place identity construct included five descriptions: “I feel this street is a part of me,” “This street is very special to me,” “I identify strongly with this street,” “I am very attached to this street,” and “Visiting this street says a lot about who I am.” The place dependence construct also comprised five descriptions: “The street is the best place for what I like to do,” “No other place can compare to this street,” “I get more satisfaction out of visiting this street than any other,” “I wouldn't substitute any other street for doing the type of things I do here,” and “Doing what I do here is more important to me than doing it in any other place.” A five-point Likert scale (from 1 = strongly disagree to 5 = strongly agree) was used for participants to evaluate the statements.

### Statistical Analyses

Data analyses were performed with SPSS software (SPSS 25.0 version, IBM, Armonk, NY, USA) and Amos 21 software (IBM, Armonk, NY, USA). We first obtained descriptive statistics for the demographic information and experimental variables. Then, we explored the reliability and validity of the questionnaires by obtaining Cronbach's α coefficients and performing a confirmatory factor analysis (CFA). Reliability and validity represent the accuracy and stability of a questionnaire. Cronbach's α coefficient is a commonly used measure of internal consistency for the reliability of questionnaire scales or tests, referring to how closely related a set of items are as a group. CFA is a multivariate statistical method that is used to test how well-measured items represent the number of variables. Composite reliability (CR) and average variance extracted (AVE) are two statistical indicators of CFA. Cronbach's α coefficients and CR were weighted for the reliability of the constructs. Convergent and discriminant validity were examined by calculating the AVE. Afterward, we conducted Spearman's correlation analysis and built a set of multiple regression models to study the associations between demographic characteristics, UGS components, perceived health benefits, and use behaviors. Collinearity statistics, measured by the variance inflation factor (VIF) ratio, were also used to test for multicollinearity issues among the variables. Furthermore, we performed mediation analyses to investigate whether place attachment mediates the relationship between UGS components and perceived health benefits and between UGS components and use behaviors. Path relationships and coefficients were measured by the maximum likelihood method in Amos 21 software. We assumed four causal pathways, as shown in Figure 1. To test the hypotheses, we applied the bias-corrected percentile method to test the total, direct, and indirect effects in the models and estimated the proportion mediated by place attachment. We assessed the 95% confidence intervals (95% CI) and proportions using 2,000 non-parametric bootstrap simulations.

## Results

### Demographic Characteristics

In total, 630 online questionnaires were collected. Two questionnaires were invalid because they were ineligible or incomplete, indicating a 99.68% valid response rate (the invalid response rate was 0.32%). Of the valid responses, 298 were from Guangzhou and 330 were from Shenzhen. Table 1 shows the sociodemographic characteristics of the 628 participants. The majority were female (64.49%), and a relatively high proportion (71.33%) were aged 18–34 years old. Regarding education, 69.43% reported that they had a bachelor's degree. In addition, 43.15% of participants had a monthly income of <1,500 CNY, followed by 28.82% with an income between 3,000 and 5,000 CNY. Approximately 50% reported that they resided in suburban districts, followed by 38.38% who were living in central cities.

**Table 1:** Demographic characteristics of participants (*N* = 628).

**Characteristics**	**Category**	***N***	**Percentage (%)**
Gender	Male	223	35.51
	Female	405	64.49
Age	18–34	448	71.33
	34–65	180	28.66
Education level	High school or below	164	26.11
	Bachelor's degree	436	69.43
	Master's degree or above	28	4.46
Income	<1,500	271	43.15
	1,500–3,000	70	11.15
	3,000–5,000	181	28.82
	5,000–10,000	58	9.24
	>10,000	48	7.64
Place of residence	Central cities	241	38.38
	Suburban districts	309	49.20
	Exurban districts	78	12.42

### Preliminary Analyses

Tables 2, 3 report the descriptive statistics, reliability and validity, correlations, and discriminant validity analysis between the variables used in the regression and mediation models, including the means, standard deviations, loading values, Cronbach's α values, CR values, and AVE and its square root values. For the UGS components, the average scores for all the variables were higher than 3 on a five-point Likert scale (*M* = 3.29–3.55). For use behaviors, the mean scores for the frequency of visits, duration of visits, and activity intensity were 2.76 ± 1.27, 2.11 ± 0.94, and 2.82 ± 0.89, respectively. For perceived health benefits, the average scores for the three variables were higher than 3 (*M* = 3.57–3.78). For place attachment, the mean score was 3.33 ± 0.80. The Cronbach's α values were higher than 0.8, which exceeded the threshold value of 0.7, showing high reliability. Additionally, the CR values of the variables were higher than the critical value of 0.7 (70), and the AVE values were above 0.5 (71), indicating acceptable convergent validity. Moreover, the AVE square root values shown in Table 3 for UGS components, perceived health benefits, and place attachment were all greater than their correlation coefficients, confirming the discriminant validity. The correlation analyses showed positive relationships between the variables, indicating that some mediating effects may exist. In summary, all the variables were highly reliable and valid.

**Table 2:** Descriptive statistics and reliability and validity analysis.

**Variable**	**Items**	**Mean**	**SD**	**Loading value**	**Cronbach's α**	**CR**	**AVE**	
UGS components	Green space attributes	Access	3.35	1.15	0.693	0.932	0.934	0.489
		Size	3.34	1.03	0.759			
		Type	3.34	1.03	0.732			
		Maintenance	3.53	0.98	0.652			
	Landscape characteristics	Road	3.55	0.99	0.721			
		Terrain	3.44	0.98	0.668			
		Water	3.36	1.05	0.729			
		Plants	3.44	1.03	0.747			
		Microclimate environments	3.58	0.98	0.693			
		Soundscape	3.43	1.02	0.786			
		Smellscape	3.45	0.99	0.729			
	Facilities	Amenity facilities	3.29	1.00	0.769			
		Rest facilities	3.55	0.97	0.68			
		Wayfinding	3.47	1.01	0.554			
		Garden ornaments	3.31	1.01	0.516			
	Perceived health benefits	Physical health	3.57	1.13	0.684	0.806	0.815	0.596
		Mental health	3.86	1.01	0.831			
		Social health	3.78	1.02	0.794			
Place attachment	Place identity	I feel this street is a part of me	3.15	1.09	0.794	0.931	0.932	0.579
		This street is very special to me	3.23	1.09	0.774			
		I identify strongly with this street	3.41	0.97	0.776			
		I am very attached to this street	3.27	1.02	0.840			
		Visiting this street says a lot about who I am	3.48	1.10	0.733			
	Place dependence	The street is the best place for what I like to do	3.46	0.93	0.717			
		No other place can compare to this street	3.25	0.98	0.737			
		I get more satisfaction out of visiting this street than any other	3.36	0.96	0.797			
		I wouldn't substitute any other street for doing the type of things I do here	3.43	0.96	0.791			
		Doing what I do here is more important to me than doing it in any other place	3.22	1.02	0.735			

*CR, composite reliability; AVE, average variance extracted*.

**Table 3:** Descriptive statistics, correlations, and discriminant validity among the variables.

**Variable**	**Mean**	**SD**	**1**	**2**	**3**
UGS components	3.43	0.73	**0.699**		
Perceived health benefits	3.74	0.89	0.536^**^	**0.772**	
Place attachment	3.33	0.80	0.555^**^	0.535^**^	**0.770**

** *Coefficient is significant at the 0.01 level (two-tailed). Square roots of the AVE are bolded on the diagonal. SD, standard deviation*.

### Predictors of Perceived Health Benefits and Use Behaviors

Table 4 displays the results of bivariate correlation analyses of the variables. All the UGS components (i.e., access, size, and type) were significantly and positively related to perceived health benefits (rs = 0.232–0.451, ps <0.01). However, only a few UGS components were significantly associated with the frequency, duration, and intensity of visits. For example, size, maintenance, road, and microclimate environment had a positively significant correlation with the duration of visits (rs = 0.109–0.132, ps <0.01), while amenity facilities showed a weak but significant relationship with the duration and intensity of visits. Almost all the demographic variables were significantly correlated with perceived health benefits and use behaviors to some extent. All the dependent variables were included in multiple linear regression models. In addition, the collinearity problem needed to be investigated because there were moderate to strong relationships between the independent variables.

**Table 4:** Correlation analyses.

	**1**	**2**	**3**	**4**	**5**	**6**	**7**	**8**	**9**	**10**	**11**	**12**	**13**	**14**	**15**	**16**	**17**	**18**	**19**	**20**	**21**	**22**	**23**	**24**	**25**	**26**
1. GEN	-																									
2. AGE	0.094^*^	-																								
3. EDU	−0.096^*^	−0.717^**^	-																							
4. INC	−0.187^*^	0.646^**^	−0.432^**^	-																						
5. RES	0.036	0.139^**^	−0.248^**^	−0.037	-																					
6. FRE	−0.059	0.486^**^	−0.458^**^	0.454^**^	0.002	−																				
7. DUR	−0.064	0.279^**^	−0.139^**^	0.378^**^	−0.014	0.222^**^	-																			
8. INT	−0.154^**^	0.090^*^	−0.074	0.185^**^	0.014	0.256^**^	0.212^**^	-																		
9. ACC	0.059	−0.186^**^	0.271^**^	−0.120^**^	−0.208^**^	−0.008	0.055	-0.034	-																	
10. SIZ	−0.022	−0.019	0.061	0.031	−0.101^*^	0.033	0.118^**^	0.057	0.297^**^	-																
11. TYP	0.034	−0.099^*^	0.120^**^	−0.058	−0.111^**^	−0.060	0.046	-0.006	0.442^**^	0.269^**^	-															
12. MAI	0.021	−0.001	0.053	0.054	−0.149^**^	0.040	0.124^**^	0.048	0.337^**^	0.417^**^	0.431^**^	-														
13. ROA	0.011	−0.012	0.062	0.028	−0.147^**^	0.005	0.103^*^	-0.007	0.371^**^	0.450^**^	0.413^**^	0.557^**^	-													
14. TER	0.017	−0.062	0.085^*^	−0.007	0.087^*^	−0.051	0.059	0.035	0.395^**^	0.395^**^	0.545^**^	0.576^**^	0.529^**^	-												
15. WAT	−0.075	0.119^**^	−0.058	0.119^**^	−0.066	0.082	0.106^*^	0.023	0.262^**^	0.383^**^	0.470^**^	0.502^**^	0.525^**^	0.420^**^	-											
16. PLA	−0.004	−0.123^**^	0.161^**^	−0.061	−0.135^**^	−0.064	0.059	0.018	0.348^**^	0.378^**^	0.477^**^	0.547^**^	0.540^**^	0.617^**^	0.423^**^	-										
17. MIC	0.022	0.038	0.025	0.080	−0.140^**^	0.085^*^	0.101^*^	0.006	0.353^**^	0.367^**^	0.417^**^	0.519^**^	0.511^**^	0.542^**^	0.545^**^	0.467^**^	-									
18. SOU	0.034	−0.229^**^	0.215^**^	−0.152^**^	−0.161^**^	−0.099^*^	−0.001	0.027	0.395^**^	0.365^**^	0.443^**^	0.445^**^	0.404^**^	0.498^**^	0.391^**^	0.465^**^	0.419^**^	-								
19. SME	0.069	−0.049	0.078	−0.013	−0.110^*^	−0.010	0.045	0.007	0.329^**^	0.406^**^	0.369^**^	0.534^**^	0.471^**^	0.519^**^	0.473^**^	0.486^**^	0.605^**^	0.464^**^	-							
20. AME	0.001	−0.018	−0.008	0.002	0.042	0.055	0.084	0.087^*^	0.242^**^	0.336^**^	0.462^**^	0.458^**^	0.454^**^	0.392^**^	0.435^**^	0.452^**^	0.360^**^	0.422^**^	0.368^**^	-						
21. RES	−0.011	−0.100^*^	0.075	−0.077	−0.149^**^	0.006	0.057	0.071	0.318^**^	0.355^**^	0.458^**^	0.509^**^	0.513^**^	0.550^**^	0.453^**^	0.527^**^	0.470^**^	0.448^**^	0.540^**^	0.388^**^	-					
22. WAY	0.016	−0.082	0.107^*^	−0.027	−0.104^*^	0.003	0.067	0.054	0.362^**^	0.388^**^	0.462^**^	0.592^**^	0.533^**^	0.549^**^	0.458^**^	0.558^**^	0.526^**^	0.506^**^	0.506^**^	0.533^**^	0.453^**^	-				
23. GAR	0.014	−0.013	−0.016	0.005	−0.038	−0.006	0.056	0.072	0.257^**^	0.359^**^	0.441^**^	0.381^**^	0.455^**^	0.502^**^	0.511^**^	0.496^**^	0.444^**^	0.421^**^	0.476^**^	0.467^**^	0.488^**^	0.452^**^	-			
24. PHY	−0.009	−0.228^**^	0.294^**^	−0.153^**^	−0.164^**^	−0.092^*^	0.030	−0.007	0.454^**^	0.243^**^	0.358^**^	0.404^**^	0.359^**^	0.392^**^	0.236^**^	0.409^**^	0.328^**^	0.426^**^	0.374^**^	0.289^**^	0.407^**^	0.367^**^	0.255^**^	-		
25. MEN	0.036	0.037	0.027	0.060	−0.151^**^	0.175^**^	0.079	0.057	0.350^**^	0.289^**^	0.261^**^	0.411^**^	0.321^**^	0.299^**^	0.265^**^	0.322^**^	0.392^**^	0.355^**^	0.407^**^	0.237^**^	0.334^**^	0.365^**^	0.234^**^	0.554^**^	-	
26. SOC	−0.041	0.091^*^	−0.040	0.119^**^	−0.028	0.238^**^	0.103^*^	0.095^*^	0.311^**^	0.267^**^	0.224^**^	0.349^**^	0.288^**^	0.286^**^	0.250^**^	0.280^**^	0.320^**^	0.281^**^	0.329^**^	0.240^**^	0.296^**^	0.304^**^	0.274^**^	0.537^**^	0.676^**^	-

*GEN, gender; AGE, age; EDU, education level; INC, income; CIT, city; RES, place of residence; FRE, frequency of visits; DUR, duration of visits; INT, activity intensity; ACC, access; SIZ, size; TYP, Type; MAI, maintenance; ROA, road; TER, terrain; WAT, water; PLA, plants; MIC, microclimate environments; SOU, soundscape; SME, smellscape; AME, amenity facilities; RES, rest facilities; WAY, wayfinding; GAR, garden ornaments; PHY, physical health; MEN, mental health; SOC, social health*.

* *p < 0.05*;

** *p < 0.01*.

Table 5 shows the results of the multiple linear regression. All the VIF ratios were under 4.0, implying that multicollinearity problems among the variables were eliminated (72). Regarding the perceived health benefits variables, notably, access, maintenance, and soundscape exerted a positive, significant, and relatively strong effect on people's perceived physical, mental, and social health benefits (βs = 0.125–0.203, ps <0.01). Model 1 accounted for nearly 34.1% of the variance, and the rest facilities (β = 0.101, *p* < 0.05) were significantly associated perceived physical health benefits. Model 2 explained ~28.2% of the variance. The microclimate environment (β = 0.122, *p* < 0.05) was reported to be significantly related to perceived mental health benefits. Model 3 explained nearly 19.6% of the variance. Water (β = −0.106, *p* < 0.05) was negatively associated with perceived social health benefits. In Models 1–3, all the F-statistics were at the 0.01 level; thus, all the predictors had strong explanatory power. In addition, people with higher education levels (β = 0.140, *p* < 0.01) were more likely to perceive UGSs as benefiting their physical health, perhaps because they had greater awareness of the value of visiting green spaces to improve health.

**Table 5:** Multiple regression results.

**Variable**		**Model 1**		**Model 2**		**Model 3**		**Model 4**		**Model 5**		**Model 6**	
		**(physical health)**		**(mental health)**		**(social health)**		**(frequency of visits)**		**(duration of visits)**		**(activity intensity)**	
		**Standardized**	**VIF**	**Standardized**	**VIF**	**Standardized**	**VIF**	**Standardized**	**VIF**	**Standardized**	**VIF**	**Standardized**	**VIF**
		**coefficients**		**coefficients**		**coefficients**		**coefficients**		**coefficients**		**coefficients**	
Demographic variables	Gender	−0.004	1.094	0.021	1.094	−0.051	1.094	−0.054	1.094	−0.044	1.094	−0.107^**^	1.094
	Age	−0.074	2.323	0.043	2.323	0.076	2.323	0.221^**^	2.323	0.196^**^	2.323	0.044	2.323
	Education level	0.140^**^	1.911	−0.037	1.911	−0.054	1.911	−0.270^**^	1.911	0.089	1.911	−0.063	1.911
	Income	−0.001	1.515	0.058	1.515	0.063	1.515	0.174^**^	1.515	0.212^**^	1.515	0.106^*^	1.515
	Place of residence	−0.024	1.220	−0.042	1.220	0.064	1.220	−0.071	1.220	0.012	1.220	−0.009	1.220
Green space attributes	Access	0.203^**^	1.505	0.153^**^	1.505	0.154^**^	1.505	0.152^**^	1.505	0.083	1.505	0.047	1.505
	Size	−0.039	1.455	0.077	1.455	0.056	1.455	0.063	1.455	0.089	1.455	0.071	1.455
	Type	0.021	1.995	−0.025	1.995	−0.042	1.995	−0.061	1.995	0.063	1.995	−0.059	1.995
	Maintenance	0.147^**^	2.366	0.184^**^	2.366	0.153^**^	2.366	0.019	2.366	0.021	2.366	−0.001	2.366
Landscape characteristics	Road	−0.007	2.090	0.009	2.090	0.009	2.090	−0.055	2.090	0.032	2.090	−0.079	2.090
	Terrain	0.035	2.584	−0.086	2.584	−0.037	2.584	−0.115^*^	2.584	−0.033	2.584	0.036	2.584
	Water	−0.069	2.073	−0.089	2.073	−0.106^*^	2.073	−0.028	2.073	−0.013	2.073	−0.054	2.073
	Plants	0.083	2.216	0.033	2.216	0.009	2.216	−0.032	2.216	0.042	2.216	−0.007	2.216
	Microclimate Environments	0.028	2.220	0.122^*^	2.220	0.075	2.220	0.108^*^	2.220	0.022	2.220	−0.037	2.220
	Soundscape	0.125^**^	1.881	0.160^**^	1.881	0.141^**^	1.881	−0.041	1.881	−0.008	1.881	−0.064	1.881
	Smellscape	0.043	2.134	0.087	2.134	0.026	2.134	−0.048	2.134	−0.046	2.134	−0.021	2.134
Facilities	Amenity facilities	0.041	1.956	0.005	1.956	0.030	1.956	0.056	1.956	−0.029	1.956	0.109^*^	1.956
	Rest facilities	0.101^*^	2.126	0.076	2.126	0.080	2.126	0.051	2.126	0.003	2.126	0.064	2.126
	Wayfinding	0.006	2.262	0.044	2.262	0.022	2.262	0.054	2.262	−0.028	2.262	0.071	2.262
	Garden ornaments	−0.051	2.033	−0.041	2.033	0.064	2.033	−0.031	2.033	−0.017	2.033	0.031	2.033
Adjusted *R*^2^		0.341		0.282		0.196		0.277		0.105		0.036	
*R* ^2^		0.362		0.305		0.222		0.300		0.133		0.067	
*F*-statistic		17.202^**^		13.339^**^		8.657		12.985		4.672		2.163	

*Independent variable—perception of green space components for health promotion*.

*VIF, variance inflation factor*.

* *p < 0.05*;

** *p < 0.01*.

As shown in Models 4–6, almost none of the UGS components were significantly related to the use behavior variables, except for the amenity facilities, combined with the results of the correlation analysis. Models 4 and 5 explained ~27.7 and 10.5% of the variance, respectively, while the adjusted *R*^2^-value for Model 6 was <0.04. Therefore, although amenity facilities (β = 0.109, *p* < 0.05) were found to be positively significantly correlated with activity intensity, the relationships between them were so weak that they were almost non-existent. Moreover, in contrast to perceived health benefits, several demographic variables appeared statistically significant. For instance, age (β = 0.221, *p* < 0.01), education level (β = −0.270, *p* < 0.01), and income (β = 0.174, *p* < 0.01) showed a significant influence on the frequency of visits. In summary, use behaviors may be more likely related to individual situations other than UGS components.

### Mediation Effects of Place Attachment

As shown in Table 6, Models 7–10 proposed in this study fit the data well. All the chi-square/df values were low and met the ideal critical criterion. To reduce the probable bias in model fit (73), we also applied several other indexes to test the model fit. The goodness-of-fit index (GFI), adjusted goodness-of-fit index (AGFI), comparative fit index (CFI) and Tucker–Lewis index (TLI) of all the models were higher than or very close to the ideal critical value of 0.9, indicating an acceptable model fit (73, 74). In addition, the root mean square error of approximation (RMSEA) values were lower than the cutoff value of 0.08, also indicating an adequate fit to the data (73).

**Table 6:** Model fit indexes.

**Model fit index**	**Ideal critical criterion**	**Acceptable value**	**Model fit statistics**			
			**Model 7**	**Model 8**	**Model 9**	**Model 10**
			**(perceived health benefits)**	**(frequency of visits)**	**(duration of visits)**	**(activity intensity)**
Chi-square/df	1–2	1–3	2.420	2.234	2.215	2.179
GFI	>0.9	>0.7	0.912	0.925	0.925	0.926
AGFI	>0.9	>0.7	0.893	0.908	0.908	0.910
RMSEA	<0.08	<0.09	0.048	0.044	0.044	0.043
CFI	>0.9	>0.7	0.956	0.963	0.963	0.965
TLI	>0.9	>0.7	0.950	0.958	0.959	0.960

*GFI, goodness-of-fit index; AGFI, adjusted goodness-of-fit index; RMSEA, root mean square error of approximation; CFI, comparative fit index; TLI, Tucker–Lewis index*.

**Figure 4:**
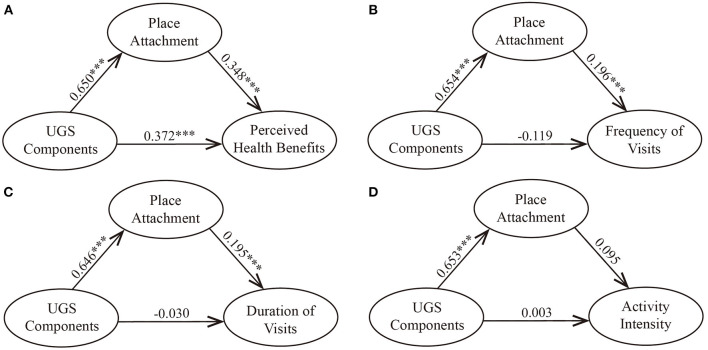
Mediation paths. **(A)** Model 7. **(B)** Model 8. **(C)** Model 9. **(D)** Model 10. ***p < 0.001.

Table 7 and Figure 4 report the results of the path analysis of the four models, including the coefficients, their significance, standard errors, and standardized coefficients. As shown, UGS components were significantly correlated with perceived health benefits (β = 0.372, *p* < 0.001), while no direct relationship was observed between UGS components and use behaviors. As predicted, UGS components were significantly related to place attachment (βs = 0.646–0.654, ps <0.001). Moreover, place attachment was also related to several outcomes, including perceived health benefits (β = 0.348, *p* < 0.001), frequency of visits (β = 0.196, *p* < 0.001), and duration of visits (β = 0.195, *p* < 0.001). In contrast, activity intensity (β = 0.095, *p* > 0.05) was not predicted by place attachment.

**Table 7:** Path analyses.

**Model**	**Path**	**Coefficient**	**SE**	**Standardized coefficient**
Model 7 (perceived health benefits)	UGS components → Perceived health benefits	0.513^***^	0.080	0.372
	UGS components → Place attachment	0.732^***^	0.072	0.650
	Place attachment → Perceived health benefits	0.427^***^	0.067	0.348
Model 8 (frequency of visits)	UGS components → Frequency of visits	−0.265	0.123	−0.119
	UGS components → Place attachment	0.741^***^	0.073	0.654
	Place attachment → Frequency of visits	0.382^***^	0.111	0.196
Model 9 (duration of visits)	UGS components → Duration of visits	−0.050	0.092	−0.030
	UGS components → Place attachment	0.726^***^	0.071	0.646
	Place attachment → Duration of visits	0.286^***^	0.082	0.195
Model 10 (activity intensity)	UGS components → Activity intensity	0.004	0.088	0.003
	UGS components → Place attachment	0.741^***^	0.072	0.653
	Place attachment → Activity intensity	0.130	0.078	0.095

*SE, standard error*.

*** *p < 0.001*.

Given the significant paths in the models, we also tested the total, direct and indirect effects (Table 8). UGS components had significant indirect effects on perceived health benefits (β = 0.226, *p* = 0.001), frequency of visits (β = 0.128, *p* = 0.001), and duration of visits (β = 0.126, *p* = 0.001). The 95% CIs of the total, direct, and indirect effects between UGS components and perceived health benefits did not include zero, nor did the indirect effects between UGS components and use behaviors, indicating that the mediating effects were significant. In summary, these findings suggest that place attachment partially mediates the relationship between UGS components and perceived health benefits but fully mediates the associations between UGS components and the frequency of visits and between UGS components and the duration of visits. These results generally support our hypotheses, except for Hypothesis 4.

**Table 8:** Model effect indexes.

**Model**	**Path**	**Standardized effect**	**Point estimate**	**Bootstrapping 95% CI**	
				**Lower**	**Upper**
Model 7 (perceived health benefits)	UGS components → → Perceived health benefits	Total	0.598^**^	0.503	0.688
		Direct	0.372^**^	0.250	0.498
		Indirect	0.226^**^	0.149	0.312
Model 8 (frequency of visits)	UGS components → Frequency of visits	Total	0.008	−0.073	0.084
		Direct	−0.119	−0.220	−0.022
		Indirect	0.128^**^	0.063	0.208
Model 9 (duration of visits)	UGS components → Duration of visits	Total	0.096	0.005	0.191
		Direct	−0.030	−0.166	0.107
		Indirect	0.126^**^	0.046	0.224
Model 10 (activity intensity)	UGS components → Activity intensity	Total	0.065	−0.018	0.153
		Direct	0.003	−0.106	0.116
		Indirect	0.062	−0.017	0.147

*95% CI, confidence intervals*.

** *p = 0.001*.

## Discussion

### General Findings

In this study, we intended to examine the impact of UGS components on people's perception of health benefits and their use behaviors in southern Chinese megacities during the COVID-19 pandemic. Our findings provide new evidence regarding what and how environmental factors are beneficial to people's health and encourage them to visit UGSs after the pandemic outbreak. In general, the results revealed that UGS components affected the perceived health benefits. However, people's use behaviors in UGSs were more likely influenced by individual characteristics rather than UGS components. Furthermore, place attachment partially mediated the relationship between UGS components and perceived health benefits but fully mediated the association between UGS components and use behaviors during the COVID-19 pandemic.

The results of the correlation and regression analyses showing that access, maintenance, and soundscape exerted a positive, significant, and relatively strong effect on perceived health benefits are consistent with the result of previous studies before the pandemic period. With regard to green space access, numerous studies before the pandemic showed that access to nature could help reduce depression and anxiety and thus improve human health (75). Francesca et al. found that people preferred to walk to small nearby urban gardens during the COVID-19 pandemic in Italy, indicating that people's need for accessible UGSs did not disappear during the pandemic (19). Maintenance, such as maintenance of vegetation and facilities, affected people's satisfaction (14) and perceived safety (15) in UGSs in the pre-pandemic era, thus impacting perceived health benefits. It is also worth mentioning that during the pandemic, the lockdown severity of green spaces was positively related to poor mental health (76). Thus, we inferred that appropriate management measures for UGSs during the pandemic period would also have a positive impact on perceived health benefits. For soundscapes, this effect can be explained by the fact that the lockdown measures conspicuously decreased traffic noise during the pandemic while allowing birds to quickly fill the song space (77). People also paid more attention to natural sounds during this time, resulting in improvements to human health and well-being. For example, Ratcliffe reported that soundscapes may improve mood and cognitive performance (78). Likewise, Rachel et al. found that water sounds and bird sounds can improve health and positive affective outcomes, including reducing pain, heart rate, and annoyance (79). In addition, we found that rest facilities were positively related to people's perceived physical health, which is consistent with previous studies in non-pandemic periods (80). It is reasonable that rest facilities such as sufficient benches might promote walking, therefore encouraging physical activities and improving physical health. Microclimate environments are also positively correlated with perceived mental health benefits. This may be attributed to a well-designed green space with a good microclimate environment that can promote activity and enable social interaction to improve mental health. A study in Canada revealed that waterscapes can arouse spiritual inspiration, peace, and self-connection (81). However, water was significantly negatively associated with perceived social health benefits in this study, possibly because different waterscapes have various subjective effects that mainly depend on the activity context of the surrounding environment. In addition, more water often involves an environment with more mosquitoes in South China, implying a risk of infectious diseases. In addition, we found no correlation between vegetation and perceived health benefits, contrary to previous works (82). This result suggests that although places with more vegetation can offer a better experience with nature, they are not necessarily the places people prefer to visit and may not be perceived as providing better health benefits during the pandemic. Thus, individual experiences and preferences should be considered when conducting research or practice.

The findings that use behaviors may be more likely related to individual situations rather than UGS components during the COVID-19 pandemic are consistent with findings from some pre-pandemic studies (32, 83, 84) but not others (85). Prior studies have highlighted the importance of social context, influence, needs, and qualities in determining participation in leisure activities (32, 83). Specifically, education level and income are positively related to use behaviors, consistent with a relevant study in Guangzhou (84), possibly because the more educated people are, the more aware they are of the benefits and importance of green spaces. Additionally, people with higher incomes tend to have more access to green spaces. Therefore, government departments should strengthen their publicity and education on the use of green spaces. Economic factors should be considered during planning and design to promote the fairness of public green space usage. Especially during the pandemic, equity for poor people may be further decreased by restrictions. Our data also show that gender may be associated with activity intensity. In this regard, Cohen et al. found that men preferred to participate in vigorous activities more than women did (86). In addition, males are more active than females in general. However, this can be attributed to women having more restrictions, such as perceived vulnerability to the pandemic (87), on their visits to green spaces than men do. More research should focus on women's constraints in the intensity of physical activity to provide insight into how to improve women's level of intensity during the pandemic. Nevertheless, a study in England showed that some biophysical properties of green spaces rather than individual situations were significantly related to visit frequency (88) before the pandemic. However, in this study, we found only that amenity facilities were significant predictors of activity intensity. Some studies suggest that the presence of certain amenity facilities, such as playgrounds, sports courts, and paths, seemed to promote physical activity (89) in the pre-pandemic period, which may explain the relationship observed during the pandemic.

Notably, our finding that place attachment partially mediates the relationship between UGS components and perceived health benefits is in line with other studies (90, 91). First, this result supports the finding that the physical features of built environments affect place attachment by cultivating a special identity for residents (92). However, a previous study indicated that higher proportions of and more accessible green space might not improve residents' place attachment (93). Researchers believe that studies of place attachment that do not control for sociodemographic characteristics should be treated with caution as the link between objective green space and place attachment may be invalid. Thus, policymakers should be wary of suggestions that do not consider the social context of the population. Second, the results suggest that place attachment may be affecting people's perceptions of the health benefits of UGSs during the COVID-19 pandemic. People who have a stronger attachment to UGSs have a positive inclination toward their impact. This is partly because human place attachment may stem from interactions reflecting the desire to satisfy specific needs for health improvement with the environment (94). Therefore, the findings verify that the mediation effect of place attachment on the relationship between UGSs and perceived health benefits still needs to be considered during the pandemic (19). Accordingly, landscape architects or urban planners should provide visitors with opportunities associated with place attachment to obtain mental, physical, and social health benefits during the COVID-19 pandemic.

Additionally, we found that UGS components influence the frequency and duration of visits mainly through the mediating role of place attachment during the COVID-19 pandemic. This result is in line with a pre-pandemic study suggesting that place attachment has a strong influence on park utilization and behavioral tendencies (95). People's emotional connection with others and attention to place can translate into an affinity for the shared environment in which they live. Therefore, place attachment may reinforce people's desire for green space visitation. This finding also further expands the former finding that use behaviors may mainly be determined by individual situations. The impact of place attachment on individuals' self-identity may improve pro-environmental behavior in their everyday lives (96). This suggests that the more attached people are to green spaces, the more likely they are to visit. This can also be explained by the fact that during the pandemic, what people have missed the most is spending time outdoors after lockdown (19), especially visiting nearby UGSs. Furthermore, some researchers have confirmed that place attachment is tied to the frequency of visits to green spaces (97) in situations without the pandemic and restrictions. One possible interpretation of these findings is that increasing contact with physical landscapes cultivates personal meanings ascribed to pristine settings, such as escape, relaxation, and perceived social cohesion (98). However, the duration of visits is not significantly associated with UGS components, and mediation analyses suggest that it may also be related to place attachment. This relationship may arise because the length of engagement and the relationship between people and places vary depending on the purpose of visiting, and place attachment influences behavioral intention. In addition, perceived safety can significantly affect the time people spend in green spaces (99) in the non-pandemic period. After the COVID-19 pandemic, the perceived safety of public spaces should be a notable factor that affects people's UGS use behaviors (100). Likewise, we found no significant relationship between activity intensity and place attachment, although Kyle et al. found that as environmental attributes improve, users tend to participate more in activities and develop more of a sense of place attachment (101). This finding can be explained by the possibility that activity intensity may mainly depend on the socioeconomic context. In summary, the influence of place attachment on the use of UGSs should still be considered during the pandemic. In addition, although we found that place attachment fully mediates the relationships in this study, this finding does not mean that place attachment is the only mediating variable; rather, it suggests that there may be other mediating variables, such as perceived safety.

### Implications for Practice

The findings show that perceived health benefits are significantly associated with UGS components rather than with sociodemographic characteristics. Thus, to promote the perceived health benefits of UGSs during the COVID-19 pandemic, we suggest that significant predictors, specifically access, maintenance, and soundscapes, should be incorporated into the relevant decision-making process to meet the diverse and evolving needs of UGSs. For example, urban planners should pay attention to small UGSs with high accessibility near residential areas, such as pocket parks and roof gardens, especially during the COVID-19 pandemic. In addition, designers should design low-maintenance landscapes, and managers should take appropriate lockdown measures for UGSs during the pandemic. Additionally, landscape architects could include appropriate natural soundscapes, such as water sounds and bird sounds, to promote mental health. Other predictors of perceived health benefits, including water, rest facilities, and the microclimate environment, are also worth noting for green space designers and managers. On the other hand, the results suggest that people's use behaviors largely depend on their social context but are not strongly related to green spaces. Accordingly, planners and managers need to provide visitors with recreation opportunities that are suitable for different groups of people. For example, we found that age, education level, and income were strong predictors of the frequency of visits. Therefore, practitioners could create green spaces suitable for all ages, establish facilities and places for nature education, and fully consider how to improve the use of green space for vulnerable groups with different social and economic attributes. Furthermore, this study revealed that we should not overlook the mediating effect of place attachment on the relationships between UGSs and perceived health benefits and between UGSs and people's use behaviors during the COVID-19 pandemic. Thus, landscape designers could attempt to maintain residents' bonds with UGSs by promoting place attachment. For example, adding local landscape elements to new environments could effectively increase visitors' place attachment and strengthen revisit intentions (61).

### Limitations and Future Research

This study provides new insights into the factors that affect people's perception of health benefits and use behaviors in UGSs in Guangzhou and Shenzhen, China, during the COVID-19 pandemic. Specifically, the mediation analyses showed possible pathways among UGS components, place attachment, perceived health benefits, and use behaviors. However, several limitations should be acknowledged in this study. First, our findings depended solely on subjective measures rather than objective measures. The results for the UGS components, perceived health benefits, and people's use behaviors may be different from the actual situation as perception may differ from reality. Therefore, for future research, we recommend that researchers consider objective measures of people's use behaviors, such as GPS trackers (102) or mobile phone data (103). Second, online surveys may lead to sample bias because researchers cannot capture the responses of those who lack access to the internet, such as the elderly, adolescents, and those with lower incomes or who reside in remote locations. Third, we used a relatively small sample of participants in a case study of a single city, considering the dimension of the city. For future research, more survey methods should be adopted, including combining field surveys and telephone surveys and sharing the questionnaire link on many other online platforms for investigations to ensure the representativeness and sufficiency of samples. Fourth, although our mediation analysis suggested a causal effect of place attachment on perceived health benefits and people's use behaviors after 2,000 separate simulations, these findings were based on cross-sectional observations. Without longitudinal data, it is impossible to establish a true cause-and-effect relationship (104). Therefore, in the future, longitudinal studies with sophisticated statistical measures should be used to track changes in people's perceptions during the pandemic over an extended period to avoid bias caused by the nature of the research and its sample. Finally, our study examined only the mediation pathways mentioned above, and the mechanisms may be relatively limited among the constructs. Therefore, future research could test other mediation pathways, such as perceived safety as a mediator in the associations between UGSs, perceived health benefits, or people's use behaviors. Furthermore, our study suggests that future research should explore the environmental factors that affect place attachment to improve perceived health benefits and encourage people to visit green spaces during the COVID-19 pandemic.

## Conclusions

COVID-19 has caused unprecedented disruption to human health and well-being and has changed people's perceptions of UGSs worldwide. Promoting the use of UGS is a vital and effective way to improve the health of urban residents. The perceived health benefits of UGSs may reflect people's visiting intentions and influence actual health. However, little is known about the influential factors for perceived health benefits and use behaviors in UGSs during the COVID-19 pandemic. Therefore, to address this question, this study used an online questionnaire survey to explore the perceived UGS factors that influence perceived health benefits and use behaviors and to further explore the role of place attachment in these relationships. The results showed that UGS components, including access, maintenance, and soundscapes, have a significant impact on people's perceived health benefits but are less affected by sociodemographic characteristics during the pandemic. In contrast to perceived health benefits, people's use behaviors are mainly affected by their social context, such as education level and income, and are less affected by UGS components. Furthermore, we found that place attachment partially mediated the association between UGS components and perceived health benefits but fully mediated the association between UGS components and use behaviors, further explaining the regression results. These findings reveal how UGSs affect perceived health benefits and use behaviors as well as the mediating role of place attachment in relationships during the COVID-19 pandemic. The results provide scientific guidance and a basis for future theoretical research, design practice, and management of UGSs to cope with the pandemic.

## Data Availability Statement

The original contributions presented in the study are included in the article/supplementary material, further inquiries can be directed to the corresponding authors.

## Ethics Statement

Ethical review and approval were not required for the study on human participants in accordance with the local legislation and institutional requirements. The participants provided their written informed consent for participation.

## Author Contributions

CD, HS, and CC contributed to the conception and design of the study. CC and NK provided the methodology and funding acquisition. HL, WL, and YH performed the investigation, statistical analysis, and wrote the draft of the manuscript. YX, CC, and JY contributed to review and editing. All authors contributed to manuscript revision, read, and approved the submitted version.

## Funding

This research was funded by the National Natural Science Foundation of China, Grant Numbers: 51808229 and 51908310 and by Projects of International Cooperation and Exchanges NSFC, Grant Number: 72111530208 and by the 2019 Philosophy and Social Science Foundation of Guangzhou, Grant Number: 2019GZGJ53 and by Research Project of Degree and Graduate Education Reform of Guangdong Province, Grant Number: 2019JGXM16.

## Conflict of Interest

CD and HS were employed by Guangzhou Sun & Partners Incorporation Design Co., Ltd. The remaining authors declare that the research was conducted in the absence of any commercial or financial relationships that could be construed as a potential conflict of interest.

## Publisher's Note

All claims expressed in this article are solely those of the authors and do not necessarily represent those of their affiliated organizations, or those of the publisher, the editors and the reviewers. Any product that may be evaluated in this article, or claim that may be made by its manufacturer, is not guaranteed or endorsed by the publisher.
